# Engagement features judged by excessive drinkers as most important to include in smartphone applications for alcohol reduction: A mixed-methods study

**DOI:** 10.1177/2055207618785841

**Published:** 2018-07-16

**Authors:** Olga Perski, Dario Baretta, Ann Blandford, Robert West, Susan Michie

**Affiliations:** 1 Department of Clinical, Educational & Health Psychology, University College London, UK; 2 Department of Psychology, University of Milano-Bicocca, Italy; 3 UCL Interaction Centre, University College London, UK; 4 UCL Institute of Digital Health, University College London, UK; 5 Department of Behavioural Science and Health, University College London, UK

**Keywords:** Alcohol reduction, behaviour change, digital health, engagement, mHealth, mixed-methods, smartphone apps, focus groups

## Abstract

**Objective:**

Engagement with smartphone applications (apps) for alcohol reduction is necessary for their effectiveness. This study explored (1) the features that are ranked as most important for engagement by excessive drinkers and (2) why particular features are judged to be more important for engagement than others.

**Methods:**

Two studies were conducted in parallel. The first was a focus group study with adult excessive drinkers, interested in reducing alcohol consumption using an app (*n*_groups_ = 3). Participants individually ranked their top 10 features from a pre-specified list and subsequently discussed their rankings. The second was an online study with a new sample (*n* = 132). Rankings were analysed using the intraclass correlation coefficient (ICC) to assess the level of agreement between raters for each study. Qualitative data were analysed using inductive thematic analysis.

**Results:**

There was low agreement between participants in their rankings, both in the focus groups (ICC = 0.15, 95% confidence interval (CI) = 0.03–0.38) and the online sample (ICC = 0.11, 95% CI = 0.06–0.23). ‘Personalisation’, ‘control features’ and ‘interactive features’ were most highly ranked in the focus groups. These were expected to elicit a sense of benefit and usefulness, adaptability, provide motivational support or spark users’ interest. Results from the online study partly corroborated these findings.

**Conclusion:**

There was little agreement between participants, but on average, the features judged to be most important for inclusion in smartphone apps for alcohol reduction were personalisation, interactive features and control features. Tailoring on users’ underlying psychological needs may promote engagement with alcohol reduction apps.

## Introduction

Approximately 43% of the world’s adults consume alcohol regularly.^1^ Excessive alcohol consumption is a risk factor for a wide range of physical (e.g. cirrhosis of the liver, cancer, stroke) and mental (e.g. depression, anxiety) conditions.^2–5^ Interventions designed to reduce excessive alcohol consumption, delivered face-to-face by trained healthcare professionals, are available in many countries.^6–8^ However, rising demand and pressures on national health budgets mean these services are limited and not meeting needs. With the advance of technology, behavioural support can be delivered digitally via websites, text messages or smartphone applications (apps). Smartphone apps support the delivery of behavioural support in real time,^9^ and have the potential to reach a large proportion of drinkers at a low cost per additional user. However, to benefit from smartphone apps for alcohol reduction, drinkers must engage with them.^10^ Although the precise nature of the relationship between engagement and intervention effectiveness is as yet unclear – particularly in the context of apps for alcohol reduction – low engagement with health apps is typically observed.^11–13^ Although many users download and try health apps, engagement is typically not sustained for more than a few occasions.^12,13^

‘Engagement’ with an app can be defined as the extent to which those who have access to it use it (e.g. how often, for how long) and the manner in which they use it (e.g. attentively).^15^ Whether a user engages with a given health app depends on its design (e.g. its content and how that content is delivered), the context in which it is used (e.g. who the users are, where and for what purpose they are using the app) and whether the app succeeds in changing particular ‘mechanisms of action’, such as users’ attitudes towards the target behaviour, skills to perform or avoid the target behaviour, or motivation to change.^15^ One plausible explanation as to why many users disengage from health apps is hence that these do not reflect users’ needs, values and circumstances.^14^

The design of health apps is often driven by the possibility of using technology, and not because the target group has expressed a need for such technology.^14^ The terms ‘co-design’ and ‘user-centred design’ are used to denote design processes in which potential users influence whether and how a design takes shape.^17^ The user-centred design process typically involves several iteratively executed stages of development, including a needs and requirements analysis, prototyping (i.e. building an early version of the software) and usability testing.^18^ Although few direct comparisons of health apps designed with and without user involvement have been made (but see DeSmet et al.^19^ for a meta-analysis of serious games designed with and without user involvement), user-centred design activities may help clarify the needs and preferences that have to be met for a particular digital intervention to be engaged with by the target group.^14,20–22^ Approaches to identifying user needs include contextual inquiry or ethnography, which can be used to identify the key issues faced by the target group, and qualitative interviews or focus groups, which can be used to identify potential users’ goals, needs and ideas for design.^23^ When an initial prototype has been developed, usability testing can shed light on how the app can be refined to better meet users’ needs.

Several smartphone apps that target alcohol reduction in adult populations have recently been developed, with different degrees of user involvement and different approaches to gathering user data. To the authors’ knowledge, the Location-Based Monitoring and Intervention System for Alcohol Use Disorders was one of the first smartphone apps designed to support adults who meet the American Psychiatric Association’s Diagnostic and Statistical Manual of Mental Disorders criteria for alcohol use disorders (AUDs) and included educational materials, feedback on alcohol consumption, advice on problem solving and craving management strategies, location-triggered alerts and advice on behaviour substitution.^24,25^ Users participating in a 6-week pilot study were asked to provide feedback on the app’s functionality and usability at the end of the trial; however, it is unclear whether their feedback was used to refine the app. The Addiction-Comprehensive Health Enhancement Support System (A-CHESS) was designed to support adult patients leaving residential treatment for AUDs and included audio-guided relaxations, location-triggered alerts and a panic button that would alert two designated contacts.^26^ Focus groups were conducted with patients, family members, criminal justice personnel and primary care physicians to gather user needs prior to the development of A-CHESS.^27^ The PartyPlanner app was designed to support alcohol reduction in university students through behavioural simulation ahead of a drinking event, and the monitoring of and tailored feedback on individuals’ estimated blood alcohol concentrations.^28^ At the end of a randomised controlled trial (RCT) of the PartyPlanner app, participants were asked to rate the app’s usability, suitability and the likelihood of recommending the app to a friend. The Alcohol Tracker app was designed to facilitate self-monitoring of alcohol consumption and included an alcohol diary, educational materials, goal setting and notifications.^29^ Although survey respondents were invited to rate the app’s perceived usefulness, the survey did not assess the app’s usability or engagement potential. The ‘CET’ app was designed by Danish psychiatrists and psychologists to deliver cue exposure therapy to adults with AUDs.^30^ User feedback on an initial version of the app was gathered through focus groups, and the app was refined accordingly prior to conducting an RCT. The Drink Less app was designed to support alcohol reduction in adults and included normative feedback, action planning, goal setting, feedback, monitoring, identity change and cognitive bias re-training.^31^ Although users were not involved in the design of the app, a usability study was conducted to gather user feedback and the app was refined prior to evaluating its components in a factorial RCT.^32^

Although many existing alcohol-reduction apps have involved users in the design process, thus increasing their engagement potential, the benefits of such user-centred design activities may be limited by involving only a small number of potential users in the design process. Although this allows researchers and designers to gain an in-depth understanding of users’ needs, insights from a small number of highly motivated participants who are willing to take part in design sessions may not generalise to other target users. For example, although community drug and alcohol service users were involved in the design of DIAMOND, a web-based alcohol intervention, few new patients recruited from the same service were willing to be randomised in a feasibility trial, mainly due to expressing a strong preference for face-to-face treatment.^33^

The present study used a mixed-methods approach, combining focus group methodology with an online study, to identify engagement features judged by excessive drinkers as most important to include in smartphone apps for alcohol reduction. We conducted in-depth focus group discussions with a small sample, in parallel with an online study with a larger sample of excessive drinkers, to address the following research questions:

1. What engagement features are ranked most highly by potential users of alcohol reduction apps?
2. What reasons do potential users give for judging particular features to be more important for engagement than others?

## Methods

### Study design

Two parallel studies were conducted. The first was a focus-group study and the second was an online study. As both methods have a number of well-known strengths and weaknesses, data sources were triangulated to address the same research questions.

Focus groups are useful for gaining an in-depth understanding of participants’ experiences, beliefs and motivations, and are particularly suitable when the interaction between participants is expected to yield additional insight into the topic of interest.^34^ Hearing about others’ experiences and views may stimulate discussion and allow participants to elaborate on ideas mentioned by other group members.^35^ However, a key weakness is that focus groups may inhibit the expression of controversial opinions due to social conformity, thus restricting the understanding of the diversity of users’ needs and preferences.^35^

Research conducted online benefits from being able to reach larger, geographically diverse samples. Hence, results from online surveys are more likely to generalise to other members of the target population than findings from focus groups. Despite these strengths, online surveys that require cognitive effort may suffer from ‘satisficing’, where respondents simply provide a satisfactory answer or randomly choose from response options.^36,37^

### Participants

#### 1. Focus groups

Drinkers were eligible to participate in one of the focus groups if they (i) were aged ≥ 18 years, (ii) lived in or near London (United Kingdom; UK), (iii) reported an Alcohol Use Disorders Identification Test (AUDIT) score of ≥ 8, indicating excessive alcohol consumption,^38^ (iv) owned an Android or iOS smartphone with internet access, (v) were interested in using a smartphone app to reduce their drinking and (vi) had previously used a health or fitness app. It was expected that participants with prior experience of using a health or fitness app would be able to more vividly imagine whether a particular feature would be important for engagement and hence generate more valid data.

Participants were recruited online through Gumtree (www.gumtree.com) and Call for Participants (www.callforparticipants.com) in addition to posters placed on central London university campuses. The recruitment materials stated that drinkers were invited to the laboratory to contribute to a focus group discussion with other participants about how to design engaging smartphone apps for alcohol reduction.

**Figure 1.**
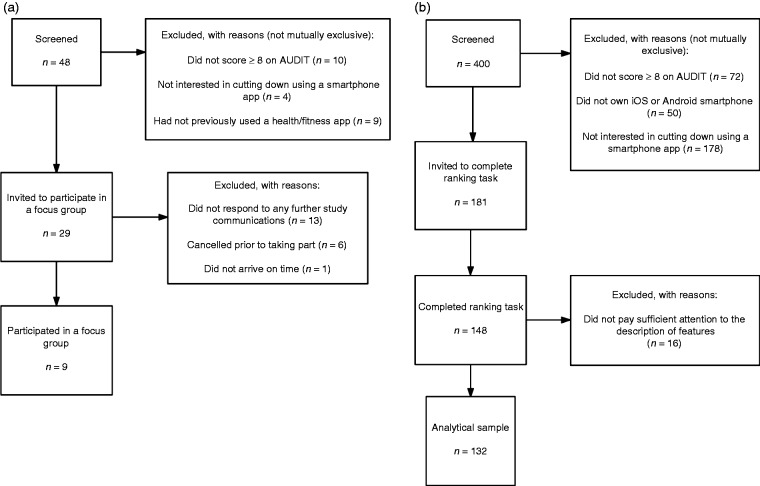
Participant flow charts for a) the focus group study, and b) the online sample.

Of the 48 participants who completed the screening questionnaire, 29 were eligible to take part. In total, 13 participants did not respond to any further study communication. Six participants cancelled prior to taking part. One participant failed to arrive on time. In total, nine participants took part in one of three focus groups, with three participants in each group (see Figure 1). The average age of participants was 30.0 years (*SD* = 10.1), 77.8% were female and 66.7% had a non-manual occupation. Participants had an average AUDIT score of 13.6 (*SD* = 3.1), indicating excessive alcohol consumption (see Table 1).

**Table 1.** Participants’ demographic and drinking characteristics.

Demographic and drinking characteristics	Focus groups, *n* (%)	Online sample, *n* (%)
Gender		
Women	7 (77.8%)	65 (49.2%)
Men	2 (22.2%)	67 (50.8%)
Age (years)		
18–24	4 (44.4%)	14 (10.6%)
25–34	3 (33.3%)	32 (24.2%)
35–44	0 (0%)	45 (34.1%)
45–54	2 (22.2%)	28 (21.2%)
55–64	0 (0%)	9 (6.8%)
65+	0 (0%)	4 (3.0%)
Occupational status		
Manual	0 (0%)	18 (13.6%)
Non-manual	6 (66.7%)	93 (70.5%)
Other	3 (33.3%)	21 (15.9%)
AUDIT, mean (*SD*)	13.6 (3.1)	16.1 (6.7)
MTSS		
1. I don’t want to cut down on drinking alcohol	1 (11.1%)	8 (6.1%)
2. I think I should cut down on drinking alcohol but I don’t really want to	1 (11.1%)	42 (31.8%)
3. I want to cut down but haven’t thought about when	4 (44.4%)	16 (12.1%)
4. I really want to cut down but I don’t know when I will	0 (0%)	10 (7.6%)
5. I want to cut down and hope to soon	1 (11.1%)	18 (13.6%)
6. I really want to cut down and intend to in the next 3 months	0 (0%)	10 (7.6%)
7. I really want to cut down and intend to in the next month	2 (22.2%)	28 (21.2%)

AUDIT = Alcohol Use Disorders Identification Test; MTSS = Motivation to Stop Scale.

#### 2. Online sample

A new sample of drinkers were eligible to participate in the online study if they met the inclusion criteria outlined above, with the exception of (ii) and (vi). Instead, participants had to reside in the UK and did not need prior experience of using a health or fitness app. As we wanted to explore generalisability, we chose to be less restrictive in the online sample. Eligible participants who did not pass a multiple-choice attention check at the end of the ranking task (i.e. “What is a professional support feature?”) were excluded from the analysis.

Participants were recruited online through Prolific Academic (www.prolific.ac). The recruitment materials invited drinkers to familiarise themselves with 16 different engagement features and rank their top 10 choices based on their likelihood of promoting engagement with apps for alcohol reduction.

Of 400 participants who completed the screening questionnaire, 181 were invited to complete the ranking task. Of these, 148 participants completed it, with 132 participants included in the analytical sample (see Figure 1). Just under half of the included participants were female (49.2%), 34.1% were aged 35–44 years, 13.6% had a manual occupation and 70.5% had a non-manual occupation. Participants had an average AUDIT score of 16.1 (*SD* = 6.7), indicating excessive alcohol consumption (see Table 1).

### Measures

Data were collected on: (1) age; (2) gender; (3) occupational status (i.e. manual, non-manual, other); 4) alcohol consumption, measured using the AUDIT; (5) interest in using a smartphone app to help cut down on alcohol (yes vs. no); and (6) motivation to cut down on drinking alcohol, measured using the Motivation to Stop Scale (MTSS).

The AUDIT is a 10-item scale that taps three domains: alcohol consumption, drinking behaviour and alcohol-related problems. There is a maximum possible score of 40, with scores between 8 and 19 indicating excessive alcohol consumption, and scores of 20 or above indicating possible dependence.^38^

The MTSS is a single-item scale with seven response options: (1) I don’t want to cut down on drinking alcohol; (2) I think I should cut down on drinking alcohol but I don’t really want to; (3) I want to cut down but haven’t thought about when; (4) I really want to cut down but I don’t know when I will; (5) I want to cut down and hope to soon; (6) I really want to cut down and intend to in the next 3 months; (7) I really want to cut down and intend to in the next month. As the majority of available tools that tap motivation to reduce alcohol are based on the Stages of Change Model,^39^ for which evidence is scarce,^40^ the MTSS was used. Although the MTSS has yet only been validated in tobacco smokers,^41^ it has been successfully employed in an observational study that estimated patterns of alcohol consumption and reduction in an English sample.^42^

### Materials

In total, 16 different engagement features, derived from a relevant systematic review,^15^ were used as stimuli (see Table 2). Feature descriptions were piloted and refined based on feedback from four independent researchers and five non-expert app users, recruited from the authors’ networks. Engagement features that have previously been found to be difficult for participants to describe verbally (e.g. aesthetics, ease of use, message tone) were not included. An experimental study design was expected to generate more valid data about how such abstract features influence engagement.^16^

**Table 2.** Engagement features used in the ranking task.

Engagement features	Descriptions and examples
Challenge features	Features that allow you to compete against yourself or against other users, such as your friends. The app might, for example, encourage you to drink one unit fewer than your friends.
Control features	Features that allow you to make choices about how to use the app. The app might, for example, allow you to choose between a few different target goals instead of having one fixed option.
Action plans to use the app	A feature that encourages you to make a plan to use the app. An example might be to make a plan to open the app as soon as you have finished your breakfast every morning.
Setting a goal to use the app	A feature that encourages you to set a goal to use the app. For example, you might be able to set a goal to use the app once a day for two weeks.
Monitoring use of the app	A feature that allows you to record your use of the app. For example, the app might allow you to manually enter how much time you have spent on it, or it might record it automatically for you.
Feedback on use of the app	A feature that allows you to view your use of the app. For example, the app might show you how many times you have opened it on each day of the week.
Credibility features	Features that make you feel you can trust the app. For example, the app might have a clear privacy policy, be endorsed by a trusted organisation, or be free from adverts.
Guidance features	Features that explain how to use the app. This might, for example, include video tutorials about how the app works.
Interactive features	Features that allow, and respond to, input from the user. This might, for example, include a game or a knowledge quiz. The direct opposite would be a static app that does not allow you to enter any information or click into any of its features, much like this piece of text!
Novelty features	Features that ensure you see or learn something new every time you open the app. This might, for example, include daily content updates (e.g. a daily fact about alcohol or a daily motivational quote).
Narrative features	The presence of a storyline. For example, the app might be set up as a game or film with a plot, where you are the main character. This might include the presence of an avatar (i.e. a virtual figure that represents you).
Personalisation	Tailoring of content according to information about you (driven by the app) or customisation of the app so it looks or acts the way you prefer (driven by you). For example, the app might tailor its content based on information you give to it (e.g. about your age, gender, level of alcohol consumption) or you might be able to change the colour and font.
Professional support features	Features that enable you to have remote contact with a healthcare professional (e.g. the opportunity to chat to a nurse or a psychologist via the app).
Social support features	Features that allow you to connect with other app users. This might, for example, include an online discussion forum or a peer-to-peer instant messenger (e.g. a ‘buddy’ system).
Reminders to use the app	Regular push notifications or text messages that remind you to use the app.
Rewards for using the app	Being rewarded for using the app. You might, for example, receive a congratulatory message or a virtual badge/coin after having opened the app for seven days in a row.

### Procedure

Interested participants read the information sheet describing the study. They subsequently provided informed consent via an online screening questionnaire, which also assessed study eligibility and collected descriptive data. The screening questionnaire was hosted by Qualtrics survey software.^43^

#### 1. Focus groups

The focus groups were conducted at University College London. Sessions lasted approximately 2 hours. Participants received a £20 gift voucher as compensation for their time. Sessions were facilitated by the first author with support from the second author.

##### Individual activity

An individual activity was first conducted to allow participants to familiarise themselves with the engagement features and elicit their attitudes to the features. The term ‘engagement’ was defined as a behaviour (e.g. how often you use the app, how much time you spend on it) and an experience (e.g. how interested you are in the app, how much attention you pay to it, how much you enjoy using it).^15^

Participants were each given a folder with Post-it Notes. Each of the 16 engagement features was described on a separate Post-it, accompanied by an illustrative example. Participants were also encouraged to think of their own examples. They were asked to rank their top 10 choices without consulting the other participants and were subsequently asked to place the Post-its with their selected features on a whiteboard, thus sharing their rankings with the group.

##### Group discussion

Participants subsequently convened to discuss their rankings. A semi-structured topic guide was used to steer the discussion (see Supplementary File 1). To gain a better understanding of why particular features were perceived as more important for engagement than others, participants were prompted to discuss the reasons for their rankings (e.g. “Can you tell me a bit more about why you ranked [insert feature here] highly?”).

#### 2. Online sample

Eligible participants were invited to complete the online ranking task in their own time on a personal computer, tablet or smartphone. The ranking task lasted for approximately 10 minutes and was hosted by Qualtrics survey software. Participants were paid £0.85 as compensation for their time. They were asked to complete the same ranking task as the focus group participants. At the end of the ranking task, participants were asked to respond to a multiple-choice attention check (described above). To gain a better understanding of why particular features were ranked more highly than others, participants were asked to respond to a free-text question about why they believed their top choice would be important for engagement.

### Data analysis

#### 1. Focus groups

Participants assigned a unique score from 1–10 to their top 10 engagement features, with 1 representing their top choice. The remaining six features were assigned a rank of 11, as the distance between these features was not expected to be meaningful. To assess the level of agreement between participants, the intraclass correlation coefficient (ICC) was estimated by means of a single measurement, absolute agreement, two-way, mixed-effects model. To assess whether some of the engagement features were, on average, ranked more highly than others, rankings were reverse scored (to aid interpretation) and descriptive statistics were calculated.

Sessions were audio recorded, transcribed verbatim and analysed using inductive thematic analysis. To inform the analysis, an interpretivist theoretical framework was used, based on the premise that the ‘lived experience’ of the individual can be captured through discussion between the researcher and participant.^44^ The thematic analysis was conducted in six phases: (i) gaining familiarity with the data, (ii) generating initial codes, (iii) searching for themes, (iv) reviewing themes, (v) defining and naming themes and (vi) producing the report.^45^ Data were coded independently by the first and second author. New inductive codes were labelled as they were identified during the coding process. Data were sometimes assigned to multiple codes. All codes that included data relating to the research questions were recorded. The first author reviewed the codes one by one, ordering the findings systematically under headings. The ordered data were reviewed and revised in discussion with the second author and were subsequently organised into themes. Disagreements were resolved through discussion. Agreement on the final themes was reached through discussion between all co-authors.

#### 2. Online sample

Participants who provided incorrect responses to the ‘attention check’ were excluded from the analysis, as incorrect responses were interpreted to suggest that participants had not paid sufficient attention to the task to provide valid data.^37^ A single measurement, absolute agreement, two-way, mixed-effects model was fitted to estimate the ICC. Rankings were reverse scored and descriptive statistics were calculated.

Responses to the free-text question about why participants believed their top choice would be important for engagement were analysed using inductive thematic analysis (described above).

### Ethical approval

Ethical approval was granted by University College London’s Departmental Research Ethics Committee (UCLIC/1213/015). Personal identifiers were removed and data were stored securely.

## Results

### 1. Engagement features ranked most highly by potential users of alcohol reduction apps

#### 1. Focus groups

**Figure 2.**
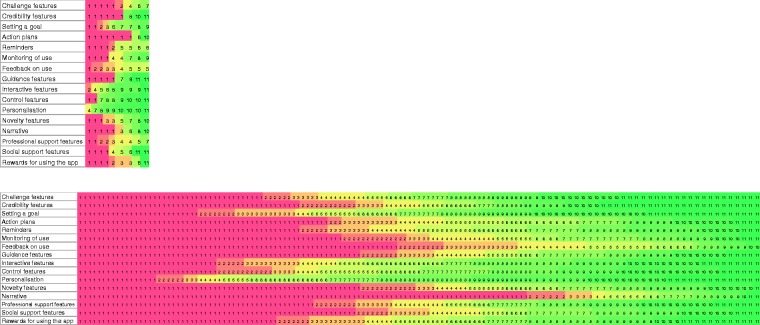
Heat maps of rankings in the focus groups (top), and in the online sample (bottom). Red, orange and yellow boxes indicate low rankings. Green boxes indicate high rankings.

There was positive but low agreement between participants (ICC = 0.15, 95% confidence interval (CI) = 0.03–0.38; see Figure 2). On average, participants ranked personalisation (*M* = 8.67, *SD* = 2.12), control features (*M* = 7.22, *SD* = 3.73) and interactive features (*M* = 7.00, *SD* = 2.92) most highly. Action plans (*M* = 2.56, *SD* = 3.24) and challenge features (*M* = 2.67, *SD* = 2.40) were judged to be the least important for engagement (see Table 3 and Figure 2).

**Table 3.** Mean rankings of the 16 engagement features in the a) focus groups (*n* = 9) and b) online sample (*n* = 132).

a) Focus groups		b) Online sample	
Engagement features	Mean (*SD*)	Engagement features	Mean (*SD*)
1. Personalisation	8.67 (*2.12*)	1. Personalisation	6.74 (*3.18*)
2. Control features	7.22 (*3.73*)	2. Setting a goal to use the app	5.97 (*3.66*)
3. Interactive features	7.00 (*2.92*)	3. Challenge features	5.56 (*3.93*)
4. Setting a goal to use the app	4.89 (*3.14*)	4. Interactive features	5.43 (*3.39*)
5. Guidance features	4.78 (*4.63*)	5. Control features	5.41 (*3.40*)
6. Social support features	4.56 (*4.13*)	6. Credibility features	4.86 (*3.99*)
7. Novelty features	4.33 (*3.35*)	7. Rewards for using the app	4.70 (*3.49*)
8. Monitoring of use	4.00 (*3.28*)	8. Professional support features	4.36 (*3.55*)
9. Credibility features	3.89 (*4.40*)	9. Reminders	4.27 (*3.20*)
10. Narrative features	3.56 (*3.54*)	10. Social support features	3.82 (*3.31*)
11. Feedback on use	3.33 (*1.50*)	11. Action plans	3.98 (*3.19*)
12. Professional support features	3.22 (*1.99*)	12. Guidance features	3.74 (*3.31*)
13. Rewards for using the app	3.22 (*3.35*)	13. Novelty features	3.66 (*3.16*)
14. Reminders	3.11 (*2.32*)	14. Monitoring of use	3.56 (*3.02*)
15. Challenge features	2.67 (*2.40*)	15. Feedback on use	2.68 (*2.33*)
16. Action plans	2.56 (*3.24*)	16. Narrative features	2.26 (*2.53*)

#### 2. Online sample

There was positive but low agreement between participants (ICC = 0.11, 95% CI =0.06–0.23; see Figure 2). On average, participants ranked personalisation (*M* = 6.74, *SD* = 3.18), setting a goal to use the app (*M* = 5.97, *SD* = 3.66) and challenge features (*M* = 5.56, *SD* = 3.93) most highly. Narrative features (*M* = 2.26, *SD* = 2.53) and feedback on use of the app (*M* = 2.68, *SD* = 2.33) were judged to be least important for engagement (see Table 3 and Figure 2).

### 2. Judgments as to why particular features are expected to be more important for engagement than others

Six themes were generated: ‘lack of trust and guidance as initial barriers’, ‘motivational support’, ‘benefit and usefulness’, ‘adaptability’, ‘sparking users’ interest’ and ‘relatedness’. Two subthemes were developed in relation to the final theme, which were labelled ‘perceived social stigma’ and ‘fear of social comparison’ (see Table 4). Additional quotations can be found in Supplementary File 2.

**Table 4.** Summary of themes and subthemes identified in a) the focus groups and b) the online sample.

Themes	Description	a) Identified in focus groups	b) Identified in online sample
1. Lack of trust and guidance as initial barriers	Features that inculcate feelings of trust and ensure the user can use the app comfortably (e.g. credibility features, guidance features) were considered more important for initial uptake than for continued engagement.	**√**	√
2. Motivational support	Features that support users’ motivation to engage with the app or to cut down on drinking (e.g. control features, rewards, setting a goal to use the app, challenge features, message tone) were expected to encourage engagement, particularly if they promote users’ independence.	**√**	**√**
3. Benefit and usefulness	Features that make users feel they are gaining something over and above *status quo* (e.g. personalisation, interactive features, novelty features, rewards) were expected to prompt engagement, particularly if they have utility ‘in real life’.	**√**	**√**
4. Adaptability	Features that allow the app to adapt its content according to the user’s level of progress or to intervene in the right moment (e.g. personalisation, interactive features, reminders) were expected to persuade the user and hence, promote engagement.	**√**	**√**
5. Sparking users’ interest	Features that grab users’ interest or provide a means of entertainment (e.g. narrative features, social support features, challenge features, interactive features, novelty features) were expected to prompt engagement.	**√**	**√**
6. Relatedness	Features that allow the user to connect with others who are in the same situation (e.g. social support features) were expected to promote engagement.	**√**	**√**
i. Perceived social stigma	Features that trigger app use in front of family and friends or connect users with close others (e.g. social support features, challenge features) were expected by some participants to elicit feelings of embarrassment and lead to disengagement.	**√**	
ii. Fear of social comparison	Features that encourage users to compete against friends or strangers (e.g. challenge features) were expected by some participants to be demoralising.	**√**	

#### 1. Lack of trust and guidance as initial barriers

Although participants expected the presence of credibility features to be necessary to decide whether to engage with the app in the first place (as such features would inculcate feelings of trust), they did not believe that credibility features would promote further engagement after having made an initial decision to download an app.

*… it wouldn’t increase my engagement behaviour. It would just be the barrier, and make sure that I would actually use it, rather than frequently use it.* P2, focus group

Similarly, the presence of guidance features was expected to aid initial app navigation, but was not expected to prompt continued engagement. If guidance was provided again later, this was expected to be annoying, as participants believed they would be capable of using the app without any further support.

*Just at the beginning of the app, when you’ve downloaded it and you’re using it for the first time, it should tell you what to do. But not every time. You don’t need guidance how to use it and where things are, because I think it would just be annoying.* P3, focus group

#### 2. Motivational support

Participants expected features that provide motivational support to be important for engagement (e.g. control features, rewards, setting a goal to use the app, challenge features). This included features that support independent decision making by, for example, allowing users to make choices about how to use the app (e.g. control features). Participants expected to feel more motivated to work towards achieving goals they had set for themselves.

*I feel that if you decide to carry out a task, you need to be in control of it, because ultimately, that’s your goal that you’re setting, and you want to have a sense of ownership or control of whatever you want to achieve. You feel more responsible for how you meet your goals.* P2, focus group

*The more I would be able to manipulate the app to be and do what I wanted or needed, for my own circumstances, the more likely I am to use it.* P16, online sample

The app’s ‘tone of voice’ or the way in which feedback was framed was expected to influence engagement. For example, feedback on drinking patterns framed in a positive manner (i.e. gain- rather than loss-framed) was expected to enhance users’ beliefs about their ability to cut down on alcohol, and hence motivate engagement with the app.

*…so that you don’t feel discouraged when you drink too much, and then you decide that, you know what, I’m just going to ignore the app and shut it off.* P8, focus group

Participants believed that setting a goal to use the app or the receipt of rewards would motivate them to return to the app. For example, virtual rewards (e.g. badges, points) were expected to automatically encourage engagement.

*It would encourage me to open the app on a daily basis.* P37, online sample

*… even if it doesn’t have practical meaning, it still works, because it’s an incentive, and it tricks your brain to thinking that you’re earning.* P3, focus group

Participants who ranked challenge features highly believed that competing against friends or other app users would help push oneself to achieve one’s targets, thus providing an important source of motivation to cut down on drinking.

*Personally, I feel if you have a community that challenges and pushes each other it encourages you to push yourself.* P47, online sample

#### 3. Benefit and usefulness

Participants believed that features that make users feel they are gaining something over and above what they already knew or felt before downloading the app would be important for engagement (e.g. personalisation, interactive features, novelty features, rewards). For example, rewards that had utility ‘in real life’ or within the app itself (e.g. unlocking novel features, shopping vouchers) were thought to be more likely to prompt engagement due to their real-world usefulness.

*Well, both of them are a kind of ‘well done for doing this’, they’re both a reward, they both make you feel a bit better. But a badge, it’s a cool fact, but it’s not the same as having vouchers, where you can go and treat yourself to something you want.* P6, focus group

Maintaining a balance between the amount of effort on the part of the user (e.g. inputting vast amounts of information) and the rewards or outputs received from the app was expected to be crucial for engagement. Participants believed they would engage with the app only if they felt they were getting something meaningful back, such as learning something new about alcohol or about themselves (e.g. through personalised feedback). They also expected that they would feel more warmly towards apps that maintained a two-way flow of communication between user and app (i.e. ‘reciprocal interactivity’).

*You’ve got to keep putting stuff in, but it’s like, when am I going to get something out of it?* P5, focus group

Participants who did not rank narrative features, action plans or goal setting to use the app highly believed that such features would distract from the main task of reducing alcohol consumption or be more effortful than rewarding.

*Well, surely the other features will make you want to use the app anyway*. P6, focus group

#### 4. Adaptability

Participants expected features that make users feel that the app adapts itself to their level of progress or intervenes in the right moment (e.g. personalisation, interactive features, reminders) to promote engagement due to inculcating the belief that the app is speaking directly to the user. Highly personalised and context-sensitive information was expected to be more persuasive than generic advice about how to drink less.

*If it’s personal to me, you just get a sense of uniqueness, and you’re like, yes, this is the best way for me to go, based on how I am right now.* P2, focus group

*Every person is an individual, so I would have more faith in the app if it felt more tailored to my personal needs.* P34, online sample

Participants also expected features that allow the app to intervene either in the right moment or pre-emptively, ‘before it is too late’, would promote engagement. For example, participants who identified as heavy drinkers expected that professional support features would encourage engagement in ‘times of crisis’.

*It would help in times of crisis to be able to be in touch with a professional, or if I needed to ask health questions related to alcoholism.* P51, online sample

However, participants who did not identify as having a problem with alcohol did not expect professional support features to encourage engagement.

*I think if I found that I had an issue with alcohol, maybe… – P9, focus group*

#### 5. Sparking users’ interest

Participants expected that the presence of features that grab users’ attention or provide a means of entertainment (e.g. interactive features, narrative features, challenge features, social support features, novelty features) would prevent boredom and hence encourage users to return to the app. The hedonistic aspect of engagement was evident in participants’ accounts, emphasising that some features are expected to be important for engagement only because they make the app more fun to use.

*An app without any interactivity would get boring very quickly, and I would probably forget about it or delete it after a while.* P72, online sample

*I do think that you need to keep people slightly entertained.* P9, focus group

Participants who ranked social support features highly believed that features that connect the user with others would draw their attention to the app and hence, promote engagement with other features.

*If you saw a message from such and such, you might be more inclined to log on and respond to them. While you’re on the app, you might use other features on it.* P6, focus group

#### 6. Relatedness

Participants who ranked social support features highly expected that such features would facilitate the receipt of non-judgmental support from other users and hence, foster a sense of relatedness.

*Being able to exchange feedback with strangers with the same goal could be supportive but non-judgemental as you will probably not know the other users.* P66, online sample

##### 

###### i. Perceived social stigma

Participants who did not rank social support or challenge features highly imagined features that trigger app use in front of family or friends or connect users with others through the app would evoke feelings of embarrassment or worry that others may think they have a problem with alcohol.

*I wouldn’t want something like: ‘Oh, why have you got that app?’* P5, focus group

###### ii. Fear of social comparison

Participants who did not rank social support or challenge features highly also pointed out that such features may have a negative effect on motivation to change due to eliciting fear of failure or worry that others are progressing quicker than oneself.

*Somebody would always do better than me, performing better on the app than me, so I’d be engaging with people who are doing better than me on the app, which might be a bit demoralising.* P4, focus group

## Discussion

### Summary of main findings

This mixed-methods study found that there was low agreement between participants concerning the importance of particular engagement features, both in the focus groups and in the online sample. In general, features judged to be most important for inclusion in smartphone apps for alcohol reduction were personalisation, control features and interactive features. These features were expected to foster a sense of benefit and usefulness, adaptability, provide motivational support or spark users’ interest. Social support and challenge features were ranked highly by a subset of participants as they were expected to foster relatedness and provide motivational support. However, another subset of potential users did not rank such features highly as they were expected to elicit social stigma or social comparison.

These findings lend support to and extend the results of prior research. First, there is previous support for the finding that personalisation is expected to promote engagement with alcohol reduction apps by inculcating the belief that the app is speaking directly to the user. Previous results have been consistent across types of study, including a formal expert consensus study^46^ and a qualitative study with potential users.^16^ This finding can be explained by the Elaboration Likelihood Model of Persuasion^47^ and the Persuasive Systems Design Model,^48^ which posit that messages tailored to users’ needs and interests have greater potential for deep (as opposed to shallow) processing. Our findings highlight two additional mechanisms through which personalisation may promote engagement. First, personalisation may help to foster a sense of benefit and usefulness. For example, encouraging users to return to the app to learn more about themselves by offering highly personalised suggestions may prevent users from feeling that they are inputting data without getting anything back. Secondly, personalisation may help to foster a sense of adaptability by supporting both user-led and reactive use. For example, participants imagined they would engage more with apps that keep up-to-date with their progress and push relevant messages to users ‘just-in-time’. Real-time message-tailoring based on current lapse risk has recently been deployed successfully in the smoking domain;^49^ this strategy also merits investigation amongst excessive drinkers. Although existing apps for alcohol reduction have incorporated location-triggered alerts,^25,26^ the utility of mood- or progress-triggered alerts is yet to be explored. A method that could be used to tailor messages in real-time is ecological momentary assessment, which has previously been used to assess drinking patterns and related cognitions and emotions.^50,51^

Secondly, previous research has emphasised the importance of features that support and develop users’ motivation.^52–54^ Participants in the present study highlighted that they would be more motivated to achieve goals they had set for themselves (i.e. ‘autonomous motivation’), suggesting this kind of motivation may be more important for engagement than motivation that arises from external contingencies (i.e. ‘controlled motivation’).^55^ However, the finding that participants also expected the receipt of rewards – which have previously been found to undermine autonomous motivation^56^ – to help them engage, begs the question as to what sources of motivation are most supportive of engagement. This should be investigated experimentally (e.g. A/B testing or a factorial experiment). It may, for example, be hypothesised that features that support users’ autonomous motivation will differentially impact on the total duration of engagement, as compared with features that support users’ controlled motivation.

Thirdly, our results suggest that users may continue to engage with alcohol reduction apps only if they are regularly provided with information or features that pique their interest. Although few studies in the alcohol domain have highlighted the importance of preventing boredom, this is not a novel idea in the digital gaming and technology literature.^57,58^ It has been argued that users have ‘non-instrumental’ needs (i.e. needs that do not serve as a means to achieve a particular aim), such as the need for stimulation or enjoyment.^59,60^ The presence of features that address these non-instrumental needs is expected to give rise to a positive user experience and hence encourage technology engagement.^60^ It has also been suggested that it may be particularly important to sustain users’ interest in the technology when they have deviated from their goals.^61^ The possibility of preventing disengagement due to relapse by providing features that meet users’ need for stimulation should therefore be explored.

Fourthly, although findings from focus groups with young adults who drink at hazardous or harmful levels indicate a strong preference for features that foster relatedness,^62^ evidence from studies with adult drinkers suggests that people typically react differently to features that connect them with friends or other users.^16^ Our results suggest that excessive drinkers may either strongly like or dislike social support features or challenge features.

The finding that there were inconsistencies in participants’ rankings begs the question as to how designers should prioritise features. By trying to satisfy everyone, we risk designing interventions that fit no one. However, as personalisation, interactive features and control features were generally preferred by excessive drinkers, a promising way forward may be to explore how these features could be embedded into alcohol reduction apps. It has been proposed that tailoring of content or features based on psychological constructs (e.g. the need for relatedness) is more effective than tailoring based on behaviour, which is in turn more effective than tailoring based on demographic characteristics.^63^ Tailoring on users’ underlying psychological needs, such as the need for relatedness, thus constitutes an important avenue for future research.

### Limitations

This study was limited by employing an abstract, cognitively demanding ranking task that may have been more suitable for a face-to-face (as opposed to an online) study context. A plausible explanation as to why goal setting to use the app was ranked highly in the online sample is that users thought this referred to goal setting for alcohol reduction. We tried to limit misunderstandings by piloting the feature descriptions, but it is possible that some participants were still confused. Although participants’ rankings should be interpreted with caution, the qualitative findings aid in the interpretation of the quantitative results.

It has been argued that users find it difficult to discuss design concepts without visual or tactile prompts, or that users are not designers.^64^ Indeed, some participants in the present study found it difficult to articulate concrete design suggestions, such as how a narrative linked to alcohol reduction would pan out. However, as we did not want to limit participants’ imagination of particular features, an abstract ranking task was deemed most suitable.

It is possible that the labels used for the engagement features may have biased participants’ attitudes. This is suggested by a study in which old adults (aged 61-94 years) agreed that a ‘falls-prevention intervention’ was a good idea, but only for people who were older or frailer than them. The authors therefore concluded that reframing the intervention as a ‘balance-training programme’ might promote uptake.^65^ In our study, labels such as ‘professional support features’ may have been perceived as too serious or irrelevant to participants’ particular situations. This was suggested by a few participants. It is therefore possible that the finding that professional support features were preferred by participants who identified as being a ‘heavy’ drinker is an artefact of the labels used.

As men tend to exhibit more alcohol-related problems than women across countries,^66,67^ the recruitment of more women than men into the focus groups constitutes a limitation. Future research should attempt to recruit a more balanced sample, with a view to exploring possible gender differences in app preferences. However, it should be noted that just over half of the online sample were male and we did not detect any differential preferences based on gender in this sample. Moreover, although the current approach to eliciting user needs provides useful information, an experimental study, in which the presence or design of particular features is manipulated, is required to test the actual impact on app engagement.

## Conclusion

There was low agreement between participants concerning the importance of particular engagement features, but on average, those judged to be most important for inclusion in smartphone apps for alcohol reduction were personalisation, interactive features and control features. This study highlights that different features may be liked and used by different users, which should be considered in the design of novel alcohol reduction apps, or the modification of existing ones. Tailoring based on users’ underlying psychological needs, such as the need for relatedness, constitutes an avenue for future research.

## Supplemental Material

Supplemental material for Engagement features judged by excessive drinkers as most important to include in smartphone applications for alcohol reduction: A mixed-methods study Click here for additional data file. Supplemental material for Engagement features judged by excessive drinkers as most important to include in smartphone applications for alcohol reduction: A mixed-methods study by Olga Perski, Dario Baretta, Ann Blandford, Robert West and Susan Michie in Digital Health

## Availability of data and materials

The full dataset supporting the conclusions of this article, containing data not already included within the article or its additional files, is available from the corresponding author on reasonable request.
