# Supplementary material for: Engagement features judged by excessive drinkers as most important to include in smartphone applications for alcohol reduction: A mixed-methods study
Source: Digit Health. 2018 Jul 16;4:2055207618785841. doi: 10.1177/2055207618785841 (PMC6048661; doi:10.1177/2055207618785841)
Supplement: Supplemental material for Engagement features judged by excessive drinkers as most important to include in smartphone applications for alcohol reduction: A mixed-methods study [file Supplemental_material.pdf]

Supplementary File 2: Additional quotations from the focus groups and the online sample

**Themes**	**Quotations**
1. Lack of trust and guidance as initial barriers	"… you'd only use it as soon as you set up the app and that's it. When you know how to work your way around the app and what features there are, and that's it. Once you know how it works, then you don't need it." – P1, focus group<br>"…it shows you around the app first, shows you what it's capable of doing, the little things it's got going on, and then it's like: "Hey! Let's start this"." – P5, focus group<br>"I would like to know that the app is from a credible source before I even contemplate using it, so this would be a must to begin." – P24, online sample
2. Motivational support	"I feel that if you decide to carry out a task, you need to be in control of it, because ultimately, that's your goal that you're setting, and you want to have a sense of ownership or control of whatever you want to achieve. You feel more responsible for how you carry out your goals." – P2, focus group<br>"I think if rewards were present I would be more likely to use the app on a regular basis." – P33, online sample<br>"I am competitive - really enjoy challenges within my network." – P45, online sample
3. Benefit and usefulness	"The information you enter, you want to get something back from it, just to increase your improvement, or whatever you want to achieve." – P2, focus group<br>"I think to feel you're getting something else out of it, it will make you think not having alcohol can still be a good thing even if that's what you want…" – P26, online sample<br>"I think that's the only way you're going to get it to work. It's got to be a two-way thing between you and the app to achieve your goals and to make sure that information that you put in is correct as well. Otherwise the whole thing is going to be a waste of time." – P1, focus group
4. Adaptability	"In the beginning, you might want to set a goal, but once the app gets more information about you, it might suggest a further goal, so it's more challenging…" – P4, focus group<br>"I think a pop-up notification could be really useful around like 6 or 5pm, which is when people get off work or off uni…" – P9, focus group<br>"Any app I engage with needs to meet my individual needs. Apps that do not have flexibility will not be used by me, regardless of their function." – P76, online sample
5. Sparking users' interest	"You want it to be interesting to you. You want it to have surprising features." – P4, focus group<br>"I feel like if there was games and quizzes to do it would encourage me to use it more as I like doing these." – P110, online task

	"It just keeps you want to engage with it, or it doesn't make you want to keep away, it makes you want to go back to it because it's actually quite good, and more features are unlocking as you're going on. You don't realise this, and this is happening…" – P5, focus group
6. Relatedness	"It just gets a lot of people in the same situation as you. You don't feel so alone." – P2, focus group<br>"Would be able to get help and support from similar people going through the same problems." – P41, online sample
i. Perceived social stigma	"I don't like the idea of putting something personal and having lots of people seeing it." – P6, focus group<br>"See, I'm quite private about things like drinking more than I want to, which is sort of where I'm up to, or I'm a lot healthier now, but there are few people that I would share that with." – P7, focus group
ii. Fear of social comparison	"…people like me that would be quite shy about my friends knowing my success or failure, day by day. I think that might be quite overwhelming…" – P7, focus group<br>"To me, it's the self-betterance. I'm trying to improve myself, so I'd rather do it just me rather than someone saying: "I used to do this..." – P5, focus group
